# Congenital Right Diaphragmatic Hernia Presenting in Adult Life: A Rare Case

**DOI:** 10.7759/cureus.31168

**Published:** 2022-11-06

**Authors:** Ramavath Hemanth Rathod, Yadavalli RD Rajan, Vijaykiran Potluri

**Affiliations:** 1 General Surgery, Siddhartha Medical College, Vijayawada, IND

**Keywords:** shortness of breath (sob), liver herniation, adult congenital diaphragmatic hernia, mesh repair, congential diaphragmatic hernia

## Abstract

A severe deformity that can occasionally be detected in infants and younger children is a congenital diaphragmatic hernia (CDH). Congenital diaphragmatic hernia is characterized by the existence of a diaphragmatic defect, most frequently to the left and posterolateral, known as Bochdalek hernia, that allows abdominal contents to herniate into the thorax. The diagnosis of this rare congenital diaphragmatic hernia is made in early childhood. When the right-sided congenital diaphragmatic hernia, which is uncommon, is asymptomatic in adulthood, the diagnosis can be challenging. We present a rare case of this condition presenting in adult life which was identified as an incidental finding when the patient was receiving treatment for dextrocardia with right bundle branch block during his teenage. To avoid entrapment and strangulation of abdominal viscera, surgical repair is advised for all adult CDH patients. Currently, numerous studies have shown that open or minimally invasive repair procedures, with or without mesh reinforcement, can be used safely and effectively. Surgical repair has been linked to minimizing morbidity and mortality, excellent long-term results, and a low rate of recurrence regardless of the method used. Reinforcement by using suture repair with mesh was preferred in this case. This case report highlights the uncommon adult right-sided Bochdalek hernia presentation and the requirement for intense clinical attention in cases that are comparable to it.

## Introduction

Diaphragmatic hernias are either congenital or acquired. The incidence of congenital diaphragmatic hernia (CDH) is 1 in 3,000-5,000 live births [1]. Right-sided diaphragmatic hernias are rare and constitute about 8-10% [2]. Left-sided lesions are more common (85%) because of the early closure of the diaphragm on the right when compared to the left and also because of the presence of the liver on the right side [3]. The congenital type occurs as a result of an improper fusion of the muscular parts of the diaphragm during development leading to herniation of intra-abdominal contents into the thoracic cavity. The acquired type occurs as a result of penetrating or blunt trauma. Most patients with congenital diaphragmatic hernia are diagnosed in early infancy or immediately post-natal period. Most adult patients are not diagnosed immediately; instead present with chronic symptoms, which include dyspnea, pleural effusion, chest pain, vague abdominal pain, postprandial fullness, nausea, and vomiting. Our patient had occasional episodes of shortness of breath while performing daily activities. Some patients are asymptomatic and are diagnosed incidentally during radiological studies [4]. We present a 42-year-old male patient who was diagnosed with a right-sided diaphragmatic hernia with dextrocardia and underwent mesh repair.

## Case presentation

**Figure 1:**
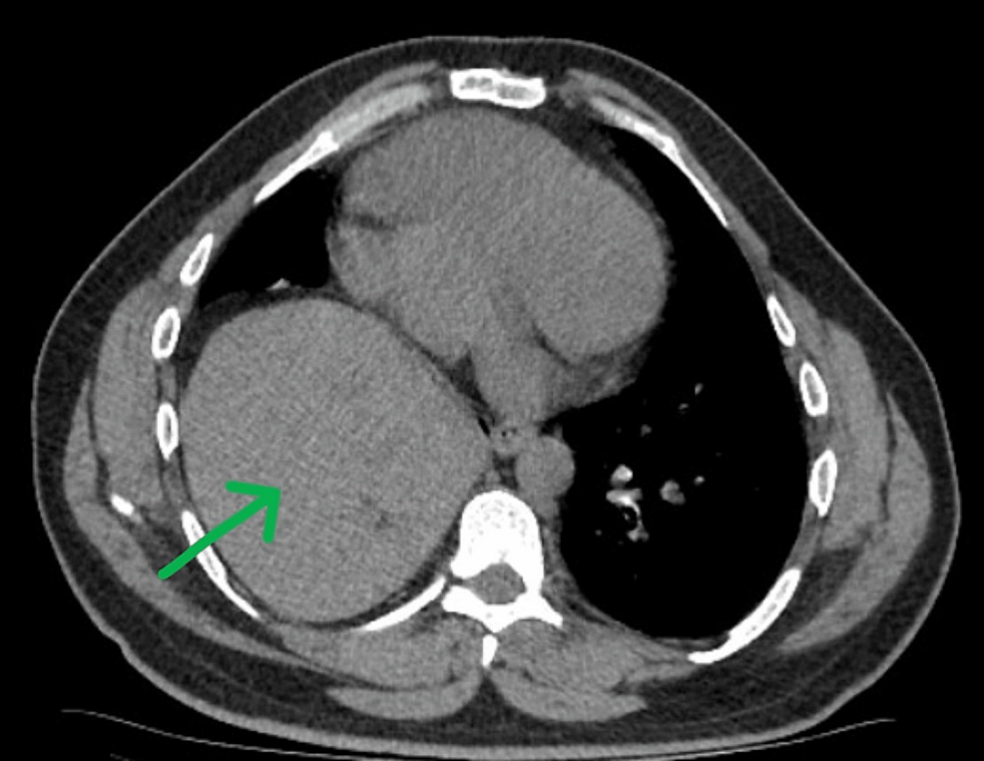
Axial section of the CT scan of the chest showing herniation of the liver into the thorax The liver (green arrow) can be seen next to the dextrocardic heart.

**Figure 2:**
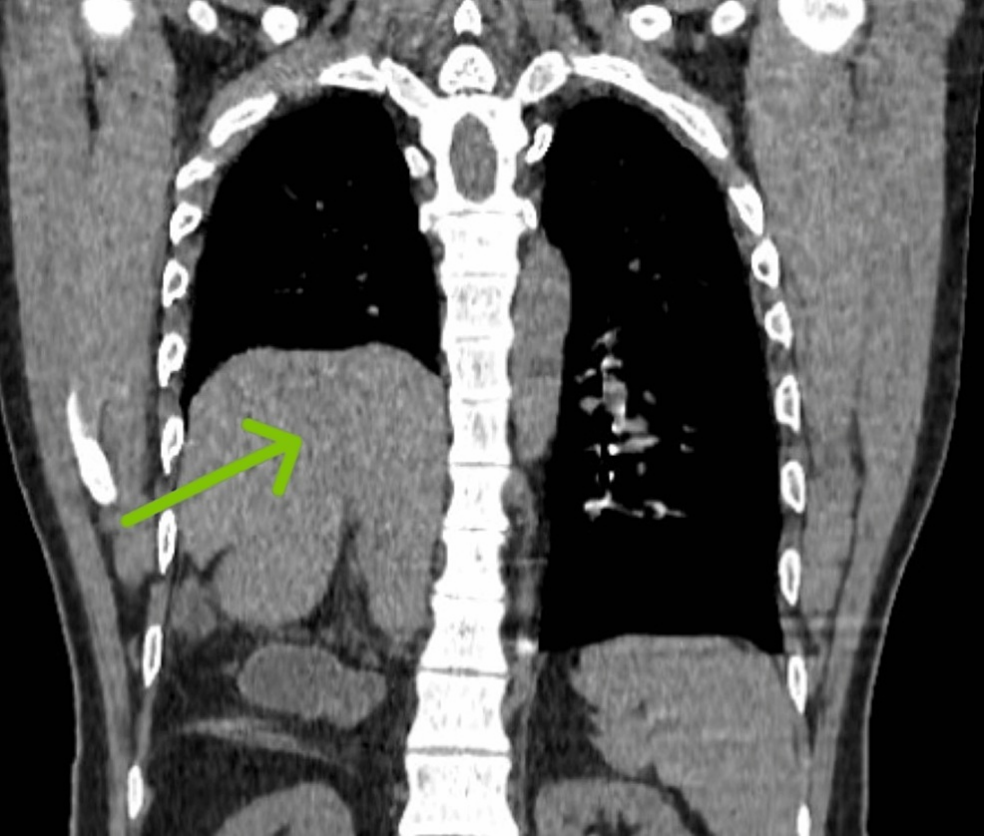
Coronal section of the CT scan of the chest showing herniation of the liver into the thorax (green arrow)

A 42-year-old male patient was referred to our center with a history of occasional episodes of shortness of breath on physical exertion. He had a previous history of chest pain during his pubertal period, where he was diagnosed with dextrocardia with right bundle branch block and right-sided diaphragmatic hernia and was not offered any treatment for diaphragmatic hernia back then. He had no history of trauma to the chest or hemoptysis. Clinical examination revealed diminished breath sounds on the right side of the lower chest region, with heart sounds heard on the right side of the chest. There was a dull note of percussion in the right chest from the fourth intercostal space. The abdominal examination was normal. Blood investigations revealed a normal complete blood picture. Chest X-ray showed homogenous opacity on the right side of the lower zone with no air shadow. CT scan of the abdomen and chest revealed an 8.3x6.2 cm defect in the posteromedial portion of the right side of the diaphragm with herniation of the right lobe of the liver into the right hemithorax, and no significant abnormality was seen in the lungs (Figures 1-2).

**Figure 3:**
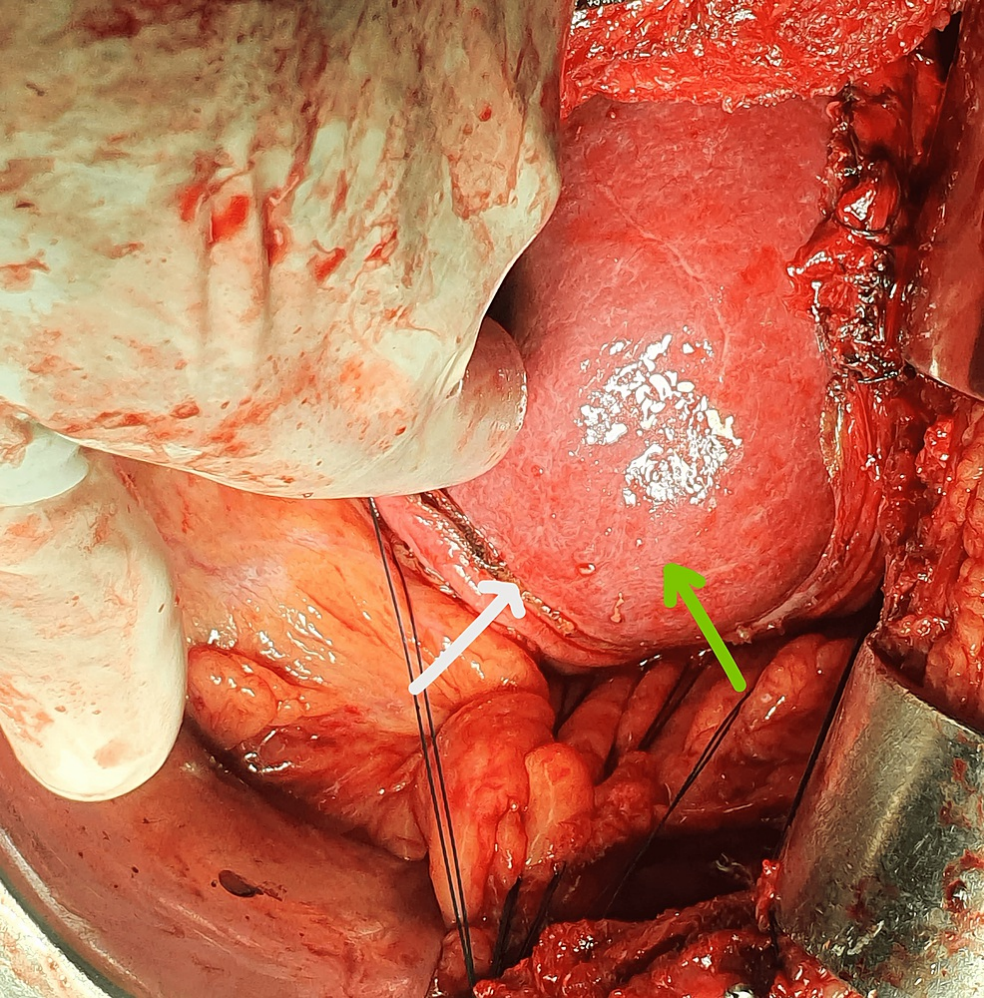
Intra-operative picture showing the herniation of the liver (green arrow) into the thorax The diaphragm (white arrow) can be seen being repaired.

Upon entering the thoracic cavity through the right posterolateral thoracotomy incision, eventration of the liver into the chest through the defect in the diaphragm was observed (Figure 3). Adhesions were found between the diaphragm and the inferior surface of the lung, which was released, and the hernial sac was excised. The liver was successfully reduced into the peritoneal cavity. The diaphragm was repaired using interrupted 2-0 polydioxanone sutures and reinforced using a 15x15 cm composite mesh. Right lung expansion was normal after the procedure. A chest tube was inserted on the right side, and the thoracotomy was closed. IV cefoperazone with sulbactam, IV metronidazole of 400 mg for five days along with IV amikacin of 500 mg for the first three days were administered. The patient was given intravenous fluids for the first three days and later shifted to oral feeds. IV paracetamol and IV tramadol were given for three days for pain management. Respiratory spirometer and chest physiotherapy have been advocated during the postoperative period. The patient was discharged on the eighth postoperative day with no recurrence in a six-month follow-up.

## Discussion

The abdominal and thoracic cavities are separated by the fusion of the septum transversum and pleuroperitoneal membranes by 8-10 weeks of life. The gastrointestinal contents come back to the abdominal cavity around the same time. Before the closure of the pleuroperitoneal canal, herniation of the abdominal contents can occur [5]. The transverse septum forms the central tendon, and the posterolateral pleuroperitoneal membranes form the muscular part of the diaphragm, which eventually fuse with the transverse septum. Failure of this fusion is the cause of a posterolateral diaphragmatic hernia [6]. Posterolateral hernias are also known as Bochdalek hernias (BH). Anterior hernias are known as Morgagni hernias. The position of diaphragmatic coronary ligaments bilaterally defines the position of the foramen of Bochdalek. This type of posterolateral hernia occurs when these ligaments fail to fuse or when they reopen. It is termed congenital when a herniation is present from the time of birth and acquired when there is an extension of intraabdominal or perirenal fat into the thorax [7]. Mortality rates are dependent on the associated degree of pulmonary hypoplasia [8]. Most of the cases that present in the later age group have a better prognosis due to a lesser degree of pulmonary hypoplasia [6]. The incidence rates are 0.02-0.03% in symptomatic patients, with females showing a higher incidence rate than males. The overall reported incidence of asymptomatic Bochdalek hernia in adults is 0.17-6% [9]. Congenital diaphragmatic hernia (CDH) presenting in adult life is a challenge to clinicians as most cases of CDH are asymptomatic or present with vague symptoms which require early diagnosis and adequate treatment.

In a study done by Brown et al., 124 articles comprising 173 patients have been reviewed in which 78% of patients had left-sided defects, 20% right-sided defects, and 2% had bilateral BH [10]. The reason why right-sided hernias are very uncommon is because of the early closure of the pleuroperitoneal membrane, and the right side of the diaphragm is buttressed by the liver. According to the literature, in adults, less than 100 cases of posterolateral hernia are documented, and less than 20 cases are right-sided [4]. The most common contents include the colon (63%), stomach (40%), omentum (39%), and small bowel (28%). The spleen, tail of the pancreas, and kidney are the unusual contents [11].

We present an unusual case of a right-sided congenital diaphragmatic hernia presenting in adult life with dextrocardia. To our best knowledge, this is the first case of right-sided congenital diaphragmatic hernia presenting in adult life with dextrocardia which was asymptomatic for both abnormalities till pubertal age. Radiological investigations were done, which included a chest radiograph that revealed an elevated right hemidiaphragm without pulmonary hypoplasia and a CT scan of the chest and abdomen, which reported an 8.3x6.2 cm defect in the posterolateral portion of the right hemidiaphragm with herniation of liver into right hemithorax with no other significant abnormality. The ideal treatment for Morgagni hernias is minimally invasive surgery with very high success rates above 90%, in contrast to Bochdalek hernias, which has high failure rates. As success rates increase with age, minimally invasive surgeries should always be considered [12]. It is controversial as to which approach is the best. Nevertheless, thoracotomy seems to be the favored approach, particularly in elective cases with larger and more complex diaphragmatic hernias [1]. Thoracotomy offers a better chance of reducing the hernia by separation from intrathoracic adhesions and direct assessment for compromised viscera before reduction [9]. Less than 5 cm defects are repaired by interrupted sutures with 2-0 polydioxanone (Reider technique). The defects of more than 5 cm in size are repaired using a tension-free mesh hernioplasty [13].

## Conclusions

Even though right-sided Bochdalek hernia (BH) is an uncommon condition, due to the rapid advances in imaging, incidental diagnosis is becoming less infrequent. To avoid future complications, BH should be managed timely, irrespective of the symptoms. It can be managed successfully by minimally invasive surgery or thoracotomy. The ideal management is not clear based on the data available; however, primary suture repair with or without mesh, whether it is synthetic or biological, is all valid.

## References

[1] Zhou Y, Du H, Che G (2014). Giant congenital diaphragmatic hernia in an adult. J Cardiothorac Surg.

[2] Slesser AA, Ribbans H, Blunt D, Stanbridge R, Buchanan GN (2011). A spontaneous adult right-sided Bochdalek hernia containing perforated colon. JRSM Short Rep.

[3] Losanoff JE, Sauter ER (2004). Congenital posterolateral diaphragmatic hernia in an adult. Hernia.

[4] Laaksonen E, Silvasti S, Hakala T (2009). Right-sided Bochdalek hernia in an adult: a case report. J Med Case Rep.

[5] Kirkland JA (1959). Congenital posterolateral diaphragmatic hernia in the adult. Br J Surg.

[6] Shenoy KR, Johri G (2013). Congenital right bochdalek hernia presenting as emergency in old age: a case report. Indian J Surg.

[7] Mullins ME, Stein J, Saini SS, Mueller PR (2001). Prevalence of incidental Bochdalek's hernia in a large adult population. AJR Am J Roentgenol.

[8] Areechon W, Reid L (1963). Hypoplasia of lung with congenital diaphragmatic hernia. Br Med J.

[9] Kohli N, Mitreski G, Yap CH, Leong M (2016). Massive symptomatic right-sided Bochdalek hernia in an adult man. BMJ Case Rep.

[10] Brown SR, Horton JD, Trivette E, Hofmann LJ, Johnson JM (2011). Bochdalek hernia in the adult: demographics, presentation, and surgical management. Hernia J Hernias Abdom Wall Surg.

[11] Machado NO (2016). Laparoscopic repair of Bochdalek diaphragmatic hernia in adults. N Am J Med Sci.

[12] Kumar A, Maheshwari V, Ramakrishnan TS, Sahu S (2009). Caecal perforation with faecal peritonitis - unusual presentation of Bochdalek hernia in an adult: a case report and review of literature. World J Emerg Surg.

[13] Kavanagh DO, Ryan RS, Waldron R (2008). Acute dyspnoea due to an incarcerated right-sided Bochdalek's hernia. Acta Chir Belg.

